# Indocyanine green excitation-emission matrix characterization: spectral shifts and application-specific spectra

**DOI:** 10.1101/2025.08.12.667954

**Published:** 2025-08-13

**Authors:** Alberto J. Ruiz, Sophie A. Lyon, Ethan P.M. LaRochelle, Kimberley S. Samkoe

**Affiliations:** a QUEL Imaging, White River Junction, VT, USA; b Dartmouth Engineering, Hanover, NH, USA

**Keywords:** indocyanine green, ICG, excitation-emission matrix, fluorescence-guided surgery, FGS, red-edge excitation shift

## Abstract

**Significance:**

Indocyanine Green (ICG) is the most widely used fluorophore in fluorescence-guided surgery (FGS), yet its spectral characteristics can significantly vary in different microenvironments. This variability impacts the design and effectiveness of fluorescence sensing systems used in medical imaging and diagnostics.

**Aim:**

Provide the first excitation-emission matrix (EEM) characterization of ICG in different microenvironments to comprehensively understand spectral shifts, including dimethyl sulfoxide (DMSO), bovine serum albumin (BSA) solutions, and 3D-printed (3DP) resin.

**Approach:**

EEMs and absorbance spectra of ICG in DMSO, BSA solutions, and 3DP resin were acquired using a CCD-based fluorescence and absorbance spectrometer. The study investigated the impact of these microenvironments and varying concentrations on ICG’s fluorescence behavior.

**Results:**

ICG in DMSO exhibited symmetric spectra across varying excitation wavelengths, confirming conventional fluorophore behavior. In contrast, ICG in BSA solution and 3DP resin showed a notable ‘rotation’ of central spectral features, indicative of red-edge excitation shifts (REES), such that fluorescence emission varied with excitation wavelength. Furthermore, varying ICG concentration measurements showed fluorescence quenching, concentration-dependent red shifts (CDRS), and disparities between absorbance and emission spectra due to inner filter effects (IFE).

**Conclusions:**

This study provides the first EEM characterization of ICG alongside the first report of REES in FGS fluorophores. These results highlight the importance of considering excitation wavelengths in spectral comparisons, illustrating that EEM data offers more comprehensive analysis than conventional fluorescence spectra collected at a single excitation wavelength. This study lays the groundwork for improved fluorophore characterizations for advancing fluorescence sensing in clinical applications, including FGS imaging.

## 1 Introduction

Understanding the spectral characteristics of a fluorophore is crucial for fluorescence sensing,^1^ including fluorescence-guided surgery (FGS).^2^ The microenvironment of a fluorophore can significantly influence its physical and chemical behaviors, with factors such as local viscosity, polarity, temperature, redox conditions, and acidic-base status playing important roles.^3^ In fluorescence imaging, the most relevant changes associated with the fluorophore’s microenvironment are the excitation-emission spectral shifts and variations in the quantum yield,^4^ since they affect the sensitivity and specificity of fluorescence sensing devices. Solvatochromism in fluorophores, which is the change in its spectral properties based on their microenvironment, generally involves shifts in absorbance or excitation-emission spectra due to solvent effects.^5,6^ As FGS continues to develop, understanding how fluorophore signals change in various microenvironments can help advance optical system design, inter-system comparisons, and facilitate the development of imaging phantoms and targets for the clinical translation of fluorescence imaging systems.^7–9^

Indocyanine green (ICG) is the most widely used fluorophore in FGS, utilized for tissue perfusion, cardiac flow indication, and lymph node mapping.^10^ Its prominence in fluorescence guidance can be attributed to its absorption and emission in the near-infrared (NIR) range, low toxicity, and extensive medical use for more than half a century.^10–13^ Understanding the variability of ICG’s spectral characteristics within *in vivo* environments can help optimize imaging system design and cross-system comparisons. This includes standard solvent effects as well as non-linear shifts, such as spectral broadening and changes in fluorescence emission at different excitation wavelengths, a phenomenon known as a red-edge excitation shift (REES).^14,15^ These non-linear effects could be particularly significant in the body’s complex environment, where proteins such as blood albumin are present.^16^

Given that system performance can affect clinical decision-making,^17^ there is a compelling need for imaging standards that can help provide ‘ground truth’ for system characterization.^7,18^ Understanding how the excitation and emission of fluorescence imaging targets can mimic *in vivo* fluorescence is essential to provide relevant one-to-one equivalence to clinical fluorescence imaging applications. Differences in fluorophore excitation and emission characteristics within different microenvironments can be quantified by acquiring its excitation-emission matrix (EEM).^19^

**Fig 1:**
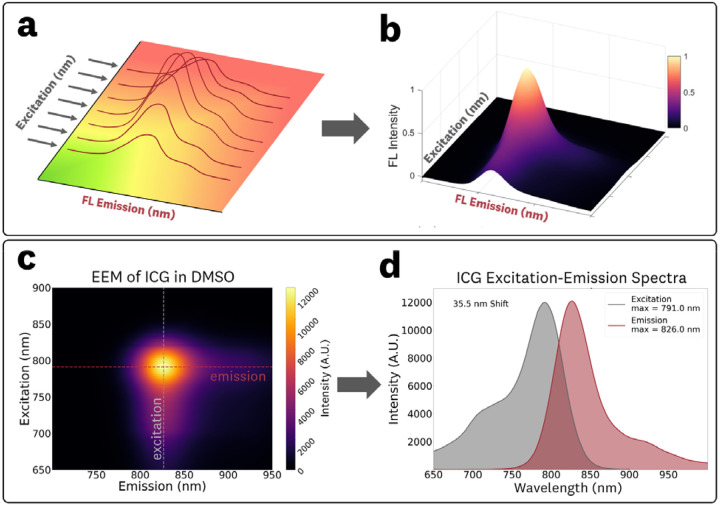
The excitation-emission matrix (EEM) of a fluorophore is generated by **(a)** acquisition of fluorescence emission spectra at various excitation wavelengths to produce the **(b)** three-dimensional EEM data set. The **(c)** EEM can be used to obtain conventional **(d)** excitation and emission spectra by isolating data along a single wavelength on the excitation or emission axis, respectively.

EEMs are three-dimensional data sets that describe the fluorescence intensity across a range of excitation and emission wavelengths. These matrices are typically acquired by collecting fluorescence emission spectra over a range of discrete excitation wavelengths (Fig 1a,b). Conceptually, they can be visualized as a series of emission spectra at different excitation wavelengths or, alternatively, as a series of excitation spectra at different emission wavelengths (Fig 1c,d). They offer insights into how fluorescence emission varies across excitation wavelengths, delivering more information than conventional excitation-emission graphs collected at a single probing wavelength. Additionally, EEMs provide the necessary data to understand phenomena such as solvachromic effects, red-edge shifts, and intensity variations across wavelengths.^15^ In FGS applications, EEMs enable the generation of system-specific fluorescence spectra based on the system’s excitation illumination. A significant limitation of EEMs is the long acquisition times associated with obtaining fluorescence spectra across various excitation wavelengths. However, recent advances in CCD-based fluorescence spectrometers have enabled the rapid acquisition of these data sets. These developments provide a promising pathway for utilizing EEMs as a superior alternative to individual spectra capture.

Here, we investigate the excitation and emission spectra changes of ICG associated with dimethyl sulfoxide (DMSO), bovine serum albumin (BSA) solution, and 3D-printed (3DP) resin microenvironment s, providing the first EEM characterization of ICG as well as the first reported instance of REES in FGS fluorophores. This study provides a comprehensive analysis of changes in spectral and fluorescence intensity within the context of the EEM, overcoming the limitations of previously reported studies,^8,9,20–24^ which focused on singular fluorescence spectral snapshots.

## 2 Methods

### 2.1 Fluorescence excitation-emission matrix acquisition

Fluorescence EEMs and absorbance spectra were collected using a CCD-based fluorescence and absorbance spectrometer (Duetta, HORIBA Scientific). This spectrometer used a xenon lamp monochromator to excite the samples, sweeping wavelengths from 650–900 nm at 1 nm steps. Fluorescence emission was detected by the CCD sensor from 600–1000 nm. All spectra were collected using a 3 nm bandpass slit size, a 0.5 nm (1 pixel) emission increment, and three measurement accumulations. The reported max fluorescence emission values are assumed to have ± 0.5 nm uncertainty, and the excitation values are assumed to have a ± 1 nm uncertainty, given the corresponding acquisition step size parameters. The uncertainty of the reported Stokes shifts is assumed to be ± 1.1 nm from adding the excitation and emission uncertainties in quadrature. A 0.75 s – 2 s integration time was used for all samples. Integration times were kept identical for comparative measurements, enabling consistent cross-sample fluorescence emission comparison. For the varying concentration measurements, the 3 μM and 10 μM samples were collected with a half integration time to avoid detector saturation such that their corresponding measured intensity counts were doubled during data processing to account for integration time differences.

The reported EEMs are calculated by subtracting the acquired data of the corresponding control (0 nM ICG) sample from the fluorescent sample. The emission spectra at a given excitation were obtained from plotting the intensity values along the emission axis of the EEM (Fig 1c,d). The excitation spectra at a “monitored” fluorescence wavelength can be derived from plotting the intensity values along the excitation axis of the EEM (Fig 1c,d).

EEMs were acquired for 1 μM and 0 μM (control) concentrations of IR-125 laser dye (Exciton Inc., 09030) in DMSO, BSA solution, and 3DP photocurable resin. IR-125 is the laser dye marketing name of ICG (sharing the same Chemical Abstracts Service number, 3599–32-4), with identical chemical, quantum yield, and fluorescence spectral characteristics.^25^ IR-125 dye was utilized for this study, rather than clinical ICG dye, due to the availability of purity specifications from the respective manufacturers. Further concentrations of IR-125 in DMSO were used to study concentration-dependent EEM effects. Each of these samples was prepared as per the respective methods sections below. Throughout the remainder of this study, the terms IR-125 and ICG are used synonymously.

### 2.2 Absorbance spectra acquisition

Absorbance spectra were collected over the 600–1000 nm range at 1 nm step increments utilizing the spectrometer silicon photodiode detector. For all samples, an integration time of 0.1 s and a band pass slit size of 3 nm were used alongside corresponding controls of DMSO, BSA, and 3DP resin as blanks. The reported max absorbance values are assumed to have a ± 1 nm uncertainty, given the corresponding acquisition step size parameters Absorbance data is reported with units of optical density (OD), where the measured transmission = 10^−OD^.

### 2.3 Data processing

**Fig 2:**
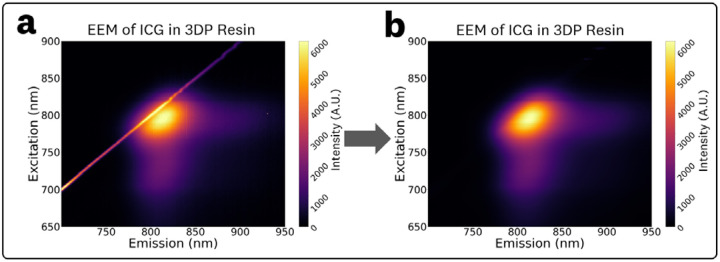
EEMs for samples with inherent scattering (3DP resin and albumin solutions) exhibit a scattering peak along the excitation-emission line **(a)** which can be corrected through the use of the RLOESS smoothing algorithm to result in the post-processed EEMs **(b)**.

The EEM and absorbance spectra were processed using robust local regression smoothing with a span of 40 nm. The particular method utilized, named RLOESS, uses robust locally weighted linear least squares regression with a 2^nd^ degree polynomial model.^26,27^ This robust smoothing allows for the elimination of outlier measurements and generally provided better peak estimations than traditional smoothing techniques (i.e. Savitzky-Golay filtering) when tested against Gaussian and skewed Gaussian fits to the spectral data. Furthermore, the robust smoothing provided the ability to eliminate scattering peaks introduced by the BSA and 3D printed resin. An example of this scattering artifact elimination by the smoothing is shown in Fig 2. RLOESS can be implemented through the use of the MATLAB function smooth() or in Python with the Pypi.org *loess* project^28,29^ that provided equivalent robust weights to the native MATLAB implementation.

### 2.4 Preparation of DMSO solution samples

EEM and absorbance measurements for the DMSO and BSA solutions were performed on standard 4.5 mL cuvettes (12×12×45 mm, 10 mm pathlength). A stock solution of 1000 μM ICG was prepared in DMSO (Sigma-Aldrich, 472301) to pre-suspend the fluorophore and minimize error during spectral comparisons.

The 1 μM ICG in DMSO cuvette sample was prepared by further diluting the 1000 μM DMSO stock to prepare 100 mL of 1 μM ICG in DMSO. 3.5 mL of this 1 μM solution was deposited into a cuvette for data collection with a corresponding control cuvette of 3.5 mL of DMSO (0 μM).

To study concentration-dependent EEM effects, an additional dilution of 100 μM was prepared from the 1000 μM DMSO stock. This 100 μM ICG stock was used to create, through serial dilution, 10 mL samples at concentrations of 10, 3, 1, 0.3, 0.1, and 0.03 μM of ICG in DMSO. Volumes of 3.5 mL of each of these solutions were deposited into cuvettes for data collection with an additional control cuvette of 3.5 mL of DMSO (0 μM ICG).

### 2.5 Preparation of BSA solution samples

The 1 μM ICG in BSA solution (44 mg/mL) cuvette sample was prepared using the 1000 μM ICG in DMSO stock, 150 g/mL BSA stock, and phosphate-buffered saline (PBS). 10x concentrated PBS (Sigma-Aldrich, P7059) was diluted tenfold with distilled water to create a 1x PBS stock appropriate for use. BSA lyophilized powder (Sigma-Aldrich, A2153) was dissolved with the 1x PBS to create a BSA stock of 150 mg/mL. 100 mL of the 1 μM ICG in BSA solution (44 mg/mL) was prepared from the 1000 μM ICG in DMSO stock, BSA stock, and 1x PBS, 3.5 mL of which was transferred to a cuvette for data collection. A corresponding BSA (44 mg/mL) control cuvette sample (0 μM ICG) was also prepared. The ICG in BSA solution was incubated at room temperature and protected from light for an hour prior to measurement. It should be noted that the 1000 μM ICG in DMSO stock solution was used in this preparation to mitigate the aggregation of dye in PBS solution, which was experimentally observed at these high concentrations. The 44 mg/mL BSA concentration was used as a representative concentration of albumin in human plasma.^30,31^

To study the effect of BSA concentration on spectral properties, 1 μM ICG cuvette samples with varying BSA concentrations were prepared using the 150 mg/mL BSA stock, a 9 μM ICG in water stock, and distilled water. The 1000 μM ICG in DMSO stock was diluted with distilled water to make a 9 μM ICG in water stock solution. BSA was diluted with distilled water to create two sets of 3.5 mL of BSA at concentrations of 100, 50, 25, 10, and 5 mg/mL. One set was kept as a control (0 μM) with the other set using 389 μL of the 9 μM ICG stock to create 3.5 mL cuvette samples of 1 μM ICG in BSA solutions of 100, 50, 25, 10, and 5 mg/mL. The samples containing ICG were incubated at room temperature and protected from light for an hour prior to measurement.

### 2.6 Preparation of 3D-printed samples

The 1 μM ICG in 3DP resin and the control (0 μM) samples were prepared using a proprietary clear photocurable resin (MML-REPC-001, QUEL Imaging) and the 1000 μM ICG in DMSO stock solution. The 3DP samples were prepared following a previously published method.^8^ In brief, 100 μL of the ICG stock was mixed into 100 mL of the clear resin and printed using layer-by-layer stereolithography printing (405 nm curing) to produce a fluorescent 3DP cuvette (12 × 12 × 40 mm^3^) to match the dimensions of the standard cuvette samples; a control cuvette (0 μM) was also printed using the same method. After printing, the cuvettes were cleaned with isopropyl alcohol and post-cured utilizing a high-radiance 385 nm light (Solarez, 88903). To minimize scattering of light, the progressively wet-sanded with distilled water using 1500, 2000, and 3000 grit silicon carbide sandpaper, followed by a two-step liquid polisher (Novus 7100). Sanding and polishing resulted in an optically clear surface, enabling data collection using the Duetta spectrometer comparable to liquid samples prepared in a standard cuvette.

## 3 Results

### 3.1 ICG in DMSO

**Fig. 3:**
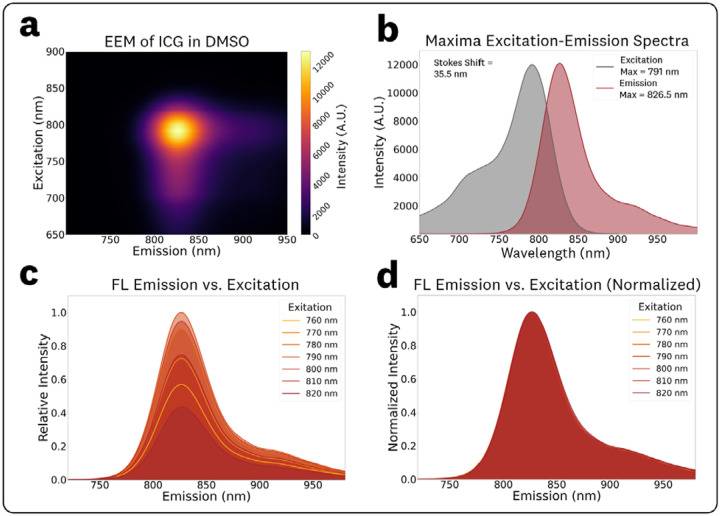
EEM and associated spectra for 1 μM ICG in DMSO: **(a)** Top-down plot of the acquired EEM. **(b)** Conventional excitation and emission spectra extracted from the EEM maxima. **(c)** Emission spectra plots at varying excitations in the 760–820 nm range at 10 nm steps to visualize intensity shifts and **(d)** normalized counterparts to visualize spectra maxima shifts.

The acquired EEM for 1 μM ICG in DMSO and associated spectra are shown in Fig 3. The EEM (Fig 3a) maxima for excitation and emission were measured as 791 nm and 826.5 nm, respectively. The associated conventional excitation and emission spectra at these maxima are plotted in Fig 3b, showing a measured Stokes shift of 35.5 nm.

To quantify spectral shifts in the fluorescence emission and intensity, spectra from varying excitation wavelength in the 760–820 nm range (10 nm steps) were extracted from the EEM data (Fig 3c,d). The identical spectral shapes of the fluorescence emission (Fig 3d) indicate there are no significant shifts in the fluorescence spectral characteristics for varying excitation wavelengths for ICG in DMSO solution. The measured maximum emissions and normalized intensities for the various excitations, reported as (excitation, emission maxima, relative emission intensity) were: (760 nm, 827.0 nm, 0.57), (770 nm, 826.5 nm, 0.72), (780 nm, 826.5 nm, 0.90), (790 nm, 826.5 nm, 1.0), (800 nm, 826.5 nm, 0.95), (810 nm, 826.5 nm, 0.75), and (820 nm, 827.0 nm, 0.43).

**Fig. 4:**
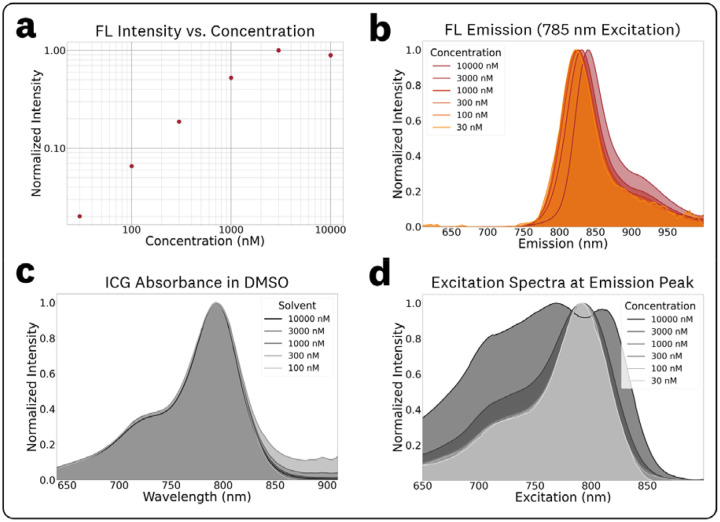
Concentration-dependent spectral effects for ICG in DMSO. **(a)** Plot of maximum fluorescence intensity vs. concentration showing a consistent increase for the 0.03 – 3 μM range with a drop-off in intensity for the 10 μM concentration. **(b)** Normalized fluorescence emission spectra at 785 nm excitation showing concentration-dependent red shifts. **(c)** Absorbance spectra show consistent spectral features over the entire concentration while the **(d)** excitation spectra show broadening and changes due to fluorescence re-absorption for the 3 μM and 10 μM concentrations.

The acquired EEMs of ICG in DMSO at varying concentrations of 0.03, 0.1, 0.3, 1.0, 3.0, and 10 μM are provided in Figure S1. These measurements showed fluorescence quenching at the 10 μM concentration (Fig 4a), concentration-dependent red shifts (CDRS) (Fig 4b), and disparities between absorbance and emission spectra due to inner filter effects (IFE) (Fig 4c,d). The log-log plot of maximum fluorescence intensity vs. concentration (Fig 4a) shows a constant increase in fluorescence emission vs. concentration for the 0.03 – 3 μM range with a drop in intensity for the 10 μM concentration. This drop in fluorescence intensity at the highest concentration is most likely attributed to both primary and secondary inner filter effects (IFE), which correspond to quenching caused by attenuation of the excitation beam and re-absorption of emitted fluorescence, respectively.^32^

To simplify discussion of concentration dependent fluorescence shifts, normalized fluorescence emission spectra at 785 nm excitation were extracted from the EEMs (Fig 4b). The corresponding measured maximum emission wavelengths and normalized intensities for these plots were: (0.03 μM, 822.0 nm, 0.020), (0.1 μM, 823.0 nm, 0.066), (0.3 μM, 824.0 nm, 0.19), (1 μM, 826.0 nm, 0.52), (3 μM, 830.5 nm, 1.0), and (10 μM, 839.5 nm, 0.89). This increase in fluorescence emission maxima (red-shift) for varying concentrations is termed CDRS, an effect that that primarily results from the re-absorption of emitted fluorescence.^32^

The measured absorbance data is plotted in Fig 4c, showing equivalent spectra from the full 0.03–10 μM concentration range. Data from the 0.03 μM sample was excluded from Fig 4c due to the low signal-to-noise ratio of its absorbance acquisition. Table S1 contains summarized OD absorbance measurements for the varying concentrations, showing no significant variation in calculated molar extinction coefficients (Table S2) for the 0.1–10 μM range. In contrast to absorbance, the excitation spectra (Fig 4d) at varying concentrations, generated from EEMs at the Fig 4b emission peaks, shows broadening and quenching for the 3 μM and 10 μM concentrations. This discrepancy between the absorbance and excitation spectra is caused by secondary IFE effects from the re-absorption of fluorescence emission at these high concentrations.^32^ with the calculated molar extinction coefficients provided in.

### 3.2 ICG in BSA Solution

**Fig. 5:**
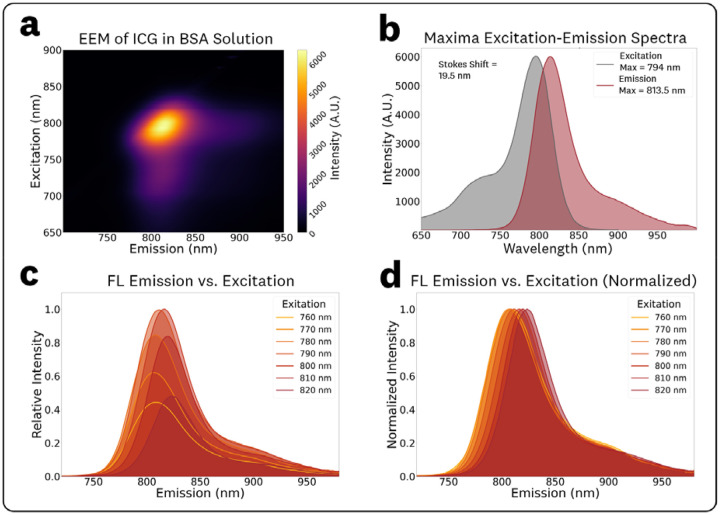
EEM and associated spectra for 1 μM ICG in BSA solution (44 mg/mL). **(a)** Top-down plot of the acquired EEM showing a ‘rotation’ of the central spectral feature indicating excitation-dependent spectral shifts. **(b)** Conventional excitation and emission spectra extracted from the EEM maxima. **(c)** Emission spectra plots at varying excitations in the 760–820 nm range at 10 nm steps to visualize intensity shifts and **(d)** normalized counterparts to visualize spectra maxima shifts.

The acquired EEM for 1 μM ICG in BSA solution (44 mg/mL) and associated spectra are shown in Fig 5. Compared to the ICG in DMSO EEM (Fig 3a), the ICG in BSA solution EEM (Fig 5a) showed a significant ‘rotation’ of the central spectra feature, indicating an excitation-dependent REES which causes significant shifts in the emission spectral characteristics for varying excitation wavelengths.^15^ The EEM maxima for excitation and emission were measured as 794.0 nm and 813.5 nm, respectively. The associated conventional excitation and emission spectra at these maxima are plotted in Fig 5b, showing a measured Stokes shift of 19.5 nm.

To quantify spectral shifts in the fluorescence emission and intensity, spectra from varying excitation wavelengths in the 760–820 nm range (10 nm steps) were extracted from the EEM data (Fig 5c,d). The fluorescence emission spectra for varying BSA concentrations (Fig 5d) indicate that there are excitation-dependent changes in the central peak wavelength but no significant changes in photon distribution (spectral broadening) for the ICG in BSA solution. The measured maximum emissions and normalized intensities for the various excitations, reported as (excitation, emission maxima, relative emission intensity) were: (760 nm, 808.0 nm, 0.44), (770 nm, 807.0 nm, 0.62), (780 nm, 807.5 nm, 0.84), (790 nm, 811.5 nm, 0.99), (800 nm, 816.0 nm, 1.0), (810 nm, 819.0 nm, 0.84), and (820 nm, 823.0 nm, 0.48). The average red-emission shift observed over the 780–820 nm excitation range was 0.39 nm of emission per nm of excitation.

**Fig. 6:**
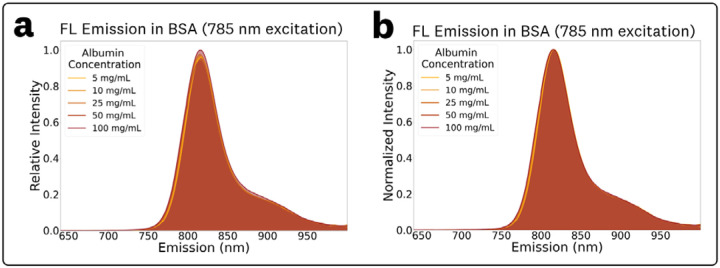
Concentration-dependent fluorescence emission measurements for the BSA solutions at 785 nm excitation for **(a)** the relative emissions to visualize changes in intensity and **(b)** normalized emissions to visualize spectral distribution changes.

The acquired ICG EEMs at different concentrations of BSA in the 5 mg/mL – 100 mg/mL range are provided in Figure S2. The EEM measurements showed no significant changes in spectral characteristics for varying BSA mg/mL concentration within the tested range, which covers the full range of biologically relevant concentrations.^30,31^ To simplify discussion of BSA concentration dependent fluorescence shifts, normalized fluorescence emission spectra at 785 nm excitation were extracted from the EEMs (Fig 6). The relative fluorescence emission plots (Fig 6a) show no significant changes in the emission intensities, and the normalized plots (Fig 6b) show no significant changes in the photon distributions of the emission. The measured maximum emissions and normalized intensities for the various concentrations of BSA were: (5 mg/mL, 816.0 nm, 0.96), (10 mg/mL, 815.5 nm, 0.96), (25 mg/mL, 815.5 nm, 0.97), (50 mg/mL, 815.0 nm, 0.94), and (100 mg/mL, 815.5 nm, 1.0).

### 3.3 ICG in 3DP Resin

**Fig. 7:**
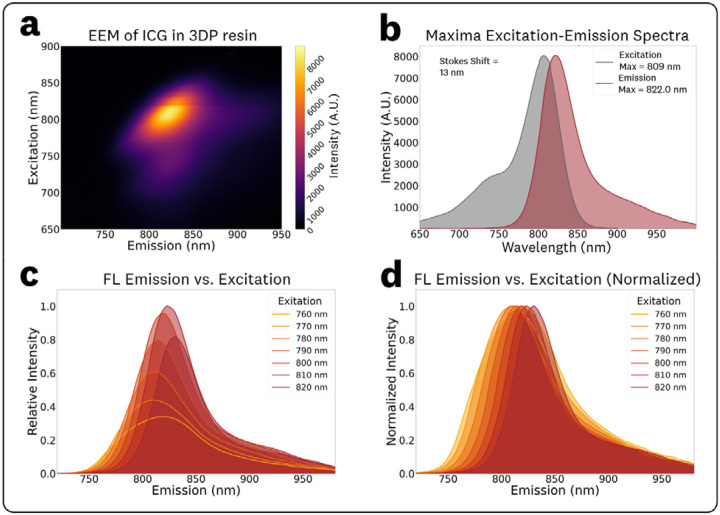
EEM and associated spectra for 1 μM ICG in 3DP resin. **(a)** Top-down plot of the acquired EEM showing a ‘rotation’ of the central spectral feature indicating excitation-dependent spectral shifts. **(b)** Conventional excitation and emission spectra extracted from the EEM maxima. **(c)** Emission spectra plots at varying excitations in the 760–820 nm range at 10 nm steps to visualize intensity shifts and **(d)** normalized counterparts to visualize spectra maxima shifts.

The acquired EEM for 1 μM ICG in 3DP resin and associated spectra are shown in Fig 7. Compared to the ICG in DMSO EEM (Fig 3a), the ICG in 3DP resin EEM (Fig 7a) showed a significant ‘rotation’ of the central spectra feature, indicating an excitation-dependent REES which causes significant shifts in the emission spectral characteristics for varying excitation wavelengths.^15^ The EEM maxima for excitation and emission were measured as 809 nm and 822.0 nm, respectively. The associated conventional excitation and emission spectra at these maxima are plotted in Fig 7b, showing a measured Stokes shift of 13 nm.

To quantify spectral shifts in the fluorescence emission and intensity, spectra from varying excitation wavelength in the 760–820 nm range (10 nm steps) were extracted from the EEM data (Fig 7c,d). The varying spectral shapes of the fluorescence emission (Fig 7d) indicate there are changes in the peak wavelength and photon distribution (spectral broadening) with excitation wavelength for ICG in 3DP resin. The measured maximum emissions and normalized intensities for the various excitations, reported as (excitation, emission maxima, relative emission intensity) were: (760 nm, 818.5 nm, 0.34), (770 nm, 809.0 nm, 0.44), (780 nm, 811.5 nm, 0.61), (790 nm, 815.0 nm, 0.80), (800 nm, 818.0 nm, 0.96), (810 nm, 823.0 nm, 1.0), and (820 nm, 830.0 nm, 0.82). The average red-emission shift observed over this excitation range was 0.45 nm per nm of excitation.

### 3.4 ICG EEM Comparison

**Fig. 8:**
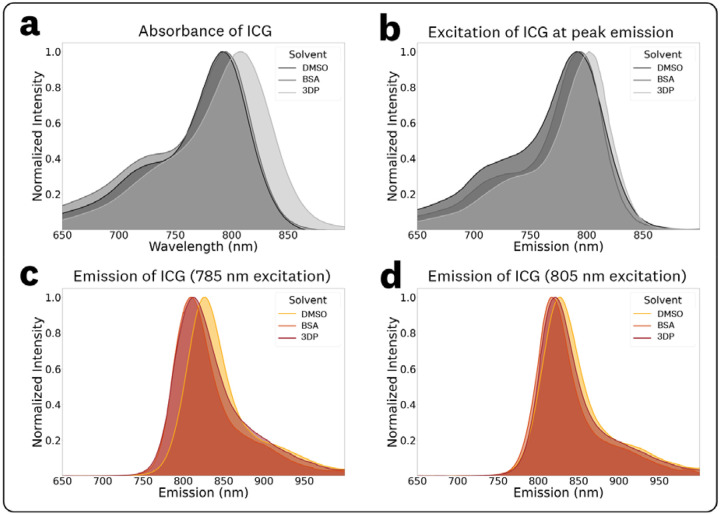
Spectral comparisons of ICG in DMSO, BSA solution (44 mg/mL), and 3DP solution: (a) Absorbance spectra, (b) excitation spectra at peak emission, (c) fluorescence emission spectra at 785 nm excitation, and (d) fluorescence emission spectra at 805 nm excitation.

The absorbance spectra, excitation at peak emission spectra, and emission spectra at 785 nm and 805 nm excitation for ICG in DMSO, BSA solution (44 mg/mL), and 3DP resin are shown in Fig 8 alongside summarized data in Table 1.

The absorbance spectra (Fig 8a) measured absorbance maxima of 792, 795, and 808 nm for DMSO, BSA solution, and 3DP resin, respectively, with similar OD for their maxima showing <5% difference in transmission (Table 1). The excitation spectra at peak emission (Fig 8b) show similar differences in peak emissions but with changing photon distributions for the BSA solution and 3DP resin. These measurements demonstrate the discrepancies between absorbance and excitation spectra caused by REES in fluorophore microenvironments.

Given that fluorescence sensing relies on fluorescence emission detection, excitation-specific spectra provide the most relevant comparisons for different microenvironments. The fluorescence emission spectra for 785 nm and 805 nm excitation are provided in Fig. 8c,d, which are two of the most common wavelengths used for ICG excitation in fluorescence imaging applications. These spectra demonstrate notable overlap between the BSA solution and 3DP resin, with almost identical peak emission intensities at the 785 nm excitation.^7^ It should be noted that despite the < 5% difference in transmittance between the three samples, the emission spectra showed ~50% decrease in fluorescence for the BSA solution and 3DP resin when compared to DMSO at 785 nm excitation (Table 1). Furthermore, the excitation-dependent fluorescence intensity and photon distributions highlight the need to consider the excitation wavelength and emission collection of the fluorescence sensing device to provide relevant comparisons within varying fluorophore microenvironments and system design parameters.

## 4 Discussion

Here we presented the first EEM measurements of ICG in DMSO, BSA solutions, and 3DP resin, allowing for a comprehensive understanding of fluorescence excitation-emission shifts in these microenvironments alongside the first reported case of REES for FGS fluorophores.

### 4.1 ICG in DMSO

The EEM for 1 μM ICG in DMSO (Fig 3) provided ‘symmetric’ spectra throughout the varying excitation wavelengths with measured emission peaks of 826.5 nm ± 0.5 nm over the 760 – 820 nm excitation range. This EEM confirms the conventional fluorophore behavior that assumes invariant spectral photon distribution of fluorescence emission with excitation wavelength.

The EEMs and absorbance at varying ICG concentrations in DMSO (Fig 4) show the effects of concentration-dependent fluorescence shifts including fluorescence quenching, CDRS, and disparities between the absorbance and excitation spectra due to IFEs. Increases in fluorophore concentration lead to a proportional increase in fluorescence emission intensity within a linear range, followed by a decrease at higher concentrations due to IFEs or fluorophore aggregation.^32,33^ The measured linear range for ICG in DMSO was 0.3 – 3 μM with the 10 μM sample showing significant quenching, emission shifts (CDRS) and distortion of the excitation spectra. The 3 μM sample exhibited minor quenching, CDRS, and excitation spectra broadening. Within the linear range, the measured EEMs (Fig S1) demonstrated no significant differences, displaying an anticipated increase in emission intensity proportional to concentration across the entire wavelength matrix. The spectral distribution of the absorbance spectra was consistent along the full concentration range, which indicates that the observed fluorescence shifts are due to IFEs and not fluorophore aggregation at these high concentrations. The photon distribution of the absorbance and excitation spectra were equivalent within the identified linear range. It is worth noting that this measured linearity range is specific to the geometry of the spectral measurement, which utilized an orthogonal excitation-emission collection with a 10 mm pathlength. These results highlight the need to understand the linearity range of a fluorophore to adequately choose concentrations for EEM and fluorescence spectra characterization.

### 4.2 ICG in BSA solution

In contrast to the ICG in DMSO EEM, the 1 μM ICG in BSA solution (Fig 5) showed a ‘rotation’ of the central spectral feature indicating the presence of REES and the dependence of emission spectra on excitation wavelength. This nonlinear effect is most likely attributed to albumin binding^16,34^ causing changes in solvent relaxation times, where the excitation red shift is observed because relaxation is not complete before fluorescence emission occurs.^15^ These results emphasize the importance of EEM fluorophore measurements to characterize spectral effects associated with different microenvironments, including REES.

EEM measurements for ICG in varying BSA concentration solutions (Fig 6, Figure S2) showed the same REES, with no significant spectral differences within the biologically-relevant albumin concentrations in the 5 – 100 mg/mL range.^30,31^ Consideration of REES fluorescence emission shifts for ICG in BSA solutions could help optimize fluorescence sensing designs including FGS imagers given the relevancy to perfusion and *in vivo* applications. The measured ICG EEM in the BSA solution (Fig 5) provides spectral information for albumin concentrations found in whole blood,^31,35^ thus serving as a good estimator of *in vivo* ICG spectra for perfusion applications. Furthermore, the invariability of the EEM within the wide albumin concentration range indicates that it might be a suitable estimator of interstitial ICG spectra.^35^

### 4.3 ICG in 3DP resin

Similarly to the ICG in BSA solution EEM, the ICG in 3DP resin EEM (Fig 7) showed a ‘rotation’ of the central spectral feature indicating the presence of REES and the dependence of emission spectra on excitation wavelength. This nonlinear effect is most likely attributed to the embedding of the fluorophore within a solid polymer matrix, causing changes in relaxation times, where the excitation red shift is observed because relaxation is not complete before fluorescence emission occurs.^15^ Given the intended use of these 3DP fluorescent materials as ‘ground-truth’ measurements, in the form of reference targets and phantoms,^7–9,18^ the presence of REES underscores the need to consider the excitation wavelength for accurate, application-specific, and system-specific characterization and comparisons.

### 4.4 ICG EEM comparison

The EEM of ICG in DMSO, BSA solution, and 3DP resin, along their corresponding fluorescence and absorption spectra (Fig 8), revealed shifts in spectral emission wavelengths and distributions, including excitation-dependent fluorescence effects in the BSA and 3DP samples. As fluorescence sensing applications predominantly focus on emission, the overlap in emission spectra at specific excitation wavelengths offers a more accurate representation of fluorescent behavior in different micro-environments. Notably, the ICG in BSA solution and 3DP resin samples displayed significant emission overlap (Fig 8c,d), particularly at excitation wavelengths commonly used in FGS devices (785–805 nm range). This indicates that the ICG in 3DP material can serve as an adequate reference for ICG in BSA solution when accounting for both system excitation and emission collection parameters.

Considering the potential shifts in both spectral distribution and peak emission wavelengths across different microenvironments and excitation wavelengths, the most accurate method for comparing fluorescence emission intensity involves integration under the curve, tailored to the specific collection parameters of the system (such as long-pass emission collection, band-pass emission collection, etc.). This dataset facilitates application and system-specific comparisons, incorporating considerations of both excitation and emission collection parameters. It is important to note that the provided EEMs are directly comparable in their reported intensities, as they were acquired using the same acquisition parameters. Although not explored in our analysis, it is worth noting that ‘weighted emission spectra’ can be generated from the provided EEMs for broadband excitation sources.

### 4.5 Future work

The presented EEM and absorbance measurements provided comprehensive insights into ICG spectral shifts in varying microenvironments but were limited to room-temperature conditions for the solvent and 3DP samples. Further insights could be gained by including whole blood in the suite of tested solvents. Although the high absorption and scattering in whole blood may pose measurement challenges, understanding REES in this context would improve the *in vivo* spectral estimations. Moreover, conducting *in vivo* fluorescence spectral measurements, which would require specialized equipment, could further understanding the predominance of albumin binding on *in-vivo* ICG EEM shifts. Understanding of temperature effects on EEMs would also provide valuable insight. Additionally, IFE corrections^36^ and spectral unmixing of EEMs could further help advance fluorophore characterization, including understanding of non-linear and aggregation effects.

As FGS targeted fluorophores become available, measuring EEM spectra for bound fluorophores that mimic the *in vivo* environment will be crucial to understanding their behavior. This is complicated further by the chemical environments of the targeted binding site. If solvent effects for a fluorophore are found to be limited, this could indicate ‘robust’ spectral properties, with no significant emission and absorption shifts observed despite changes in microenvironment. Conversely, significant spectral changes due to solvent effects would be important to identify prior to clinical application and could inform the imaging system design or further modification of the fluorophore structure.

## 5 Conclusion

This study presents the first detailed EEM characterization of ICG, including DMSO, BSA solutions, and 3DP resin microenvironments. Our research provides new insights into the spectral shifts of ICG, notably the presence of REES in FGS fluorophores. The results highlight the significant impact of different solvents and environments on the fluorescence behavior of ICG, emphasizing the necessity of accounting for these variations in fluorescence sensing applications. The observed spectral changes, dependent on excitation wavelengths and specific microenvironments, stress the importance of precise fluorophore concentration selection and a thorough understanding of spectral properties for effective biomedical imaging. Furthermore, this underscores the importance of considering excitation wavelength in spectral comparisons, showing that EEM data, which captures spectral variations across a range of excitation wavelengths, provides a more comprehensive analysis than conventional fluorescence spectra collected at a single wavelength. This study lays the groundwork for improved fluorophore characterizations to further explore these spectral shifts under varied conditions, including temperature effects and *in vivo* environments, which can help optimize fluorophore chemistry and fluorescence-based imaging system design for clinical settings.

## Supplementary Material

Supplement 1

## Figures and Tables

**Table 1:** Summarized data for the comparison of the spectral behavior of ICG in DMSO, BSA solution and 3DP resin microenvironments.

	Absorbance		Excitation		Emission (785 nm excitation)		Emission (805 nm excitation)	
	Max (nm)	OD	Max (nm)	Intensity (au)	Max (nm)	Intensity (au)	Max (nm)	Intensity (au)
**DMSO**	792	0.167	791	n/a	826	1.00	827	1.00
**Albumin**	795	0.142	794	n/a	810	0.478	817	0.538
**3DP**	808	0.158	802	n/a	813	0.487	821	0.772

## Data Availability

Data and code are available upon reasonable request.
